# Homecare workers - an untapped resource in preventing emergency department visits among older individuals? A qualitative interview study from Sweden

**DOI:** 10.1186/s12877-024-04906-5

**Published:** 2024-04-18

**Authors:** Pia Bastholm-Rahmner, Monica Bergqvist, Karin Modig, Lars L. Gustafsson, Katharina Schmidt-Mende

**Affiliations:** 1 Academic Primary Healthcare Center, Region Stockholm, Sweden; 2 Department of Laboratory Medicine, Division of Clinical Pharmacology, Karolinska Institutet, Karolinska Institutet, Stockholm, Sweden; 3 Department of Neurobiology, Care Sciences and Society, Division of Nursing, Karolinska Institutet, Stockholm, Sweden; 4 Institute of Environmental Medicine, Unit of Epidemiology, Karolinska Institutet, Stockholm, Sweden; 5 Department of Neurobiology and Care Sciences and Society, Division of Family Medicine and Primary Care, Karolinska Institutet, Stockholm, Sweden

**Keywords:** Emergency departments, Frail older adults, Homecare aids, Qualitative research, Primary care

## Abstract

**Background:**

Older individuals with functional decline and homecare are frequent visitors to emergency departments (ED). Homecare workers (HCWs) interact regularly with their clients and may play a crucial role in their well-being. Therefore, this study explores if and how HCWs perceive they may contribute to the prevention of ED visits among their clients.

**Methods:**

In this qualitative study, 12 semi-structured interviews were conducted with HCWs from Sweden between July and November 2022. Inductive thematic analysis was used to identify barriers and facilitators to prevent ED visits in older home-dwelling individuals.

**Results:**

HCWs want to actively contribute to the prevention of ED visits among clients but observe many barriers that hinder them from doing so. Barriers refer to care organisation such as availability to primary care staff and information transfer; perceived attitudes towards HCWs as co-workers; and client-related factors. Participants suggest that improved communication and collaboration with primary care and discharge information from the ED to homecare services could overcome barriers. Furthermore, they ask for support and geriatric education from primary care nurses which may result in increased respect towards them as competent staff members.

**Conclusions:**

HCWs feel that they have an important role in the health management of older individuals living at home. Still, they feel as an untapped resource in the prevention of ED visits. They deem that improved coordination and communication between primary care, ED, and homecare organisations as well as proactive care would enable them to add significantly to the prevention of ED visits.

**Supplementary Information:**

The online version contains supplementary material available at 10.1186/s12877-024-04906-5.

## Introduction

The policy of “aging-in-place” and a desire among older individuals to be cared for in their familiar surrounding has increased the number of individuals with complex care needs living at home [1–4]. Older individuals who reside at home primarily receive their social care from homecare services, with primary care (PC) serving as their principal healthcare provider [5]. Nevertheless, they frequently visit emergency departments (ED) [1–4]. At the same time, older individuals report experiencing anxiety and insecurities due to ED visits [6, 7]. Moreover, ED visits are associated with adverse outcomes such as functional decline, readmissions, revisits to the ED and mortality [8]. Older individuals utilising homecare exhibit a significantly elevated risk of readmission and mortality following an ED admission compared to those not utilising homecare [9, 10].

There has been some debate about what constitutes a preventable ED visit and there is no uniform definition of preventable ED visits for older individuals [11, 12]. Preventability may either refer to the degree of acuity after triage, the absence of subsequent hospitalisation, or acute situations that are likely to be prevented by timely and effective outpatient care as proposed in the concept of ambulatory care sensitive conditions [13]. Nevertheless, a systematic literature review concludes that one in four ED visits in frail older individuals could be prevented, for example by improved management of heart failure in PC [13]. According to older individuals themselves, their relatives, general practitioners and ED physicians, ED visits were considered partly preventable (12%, 9%, 21% and 31%, respectively) [12]. Patients tend to blame themselves when needing to seek emergency care, while healthcare providers primarily attribute the need for such care to inadequate communication among professionals, thus identifying it as a key contributing factor in preventing ED visits [12]. Other risk factors for ED visits among older individuals include perceived and/or actual poor health status and previous ED visits [14]. High numbers of preventable visits to ED may reflect deficiencies in both accessibility, continuity, and quality of care of PC [15–17]. While not all ED visits can be prevented, PC that offers prevention, early identification and continuous disease management could probably reduce reliance on ED visits among older individuals [18].

Above findings may imply that the local healthcare system rather than older individuals’ decisions to seek care is of major importance for the prevention of ED visits. To date there has been limited research on how contextual factors affect the local healthcare system and homecare services, i.e., those professionals who work closest to older individuals. In addition, there is little research on how homecare workers (HCWs) perceive care [19–21] and, in particular, how they think they may contribute to the prevention of ED visits among home-dwelling older individuals.

In Sweden, homecare services are provided by the municipality to enable older individuals to safely “age-in-place” (Box 1).

**Box 1:** Organisation of homecare services in Sweden

• Homecare services are largely funded by municipal taxes and government grants and organized locally by the 290 municipalities. The Social Service Act [22] regulates how the municipalities are to organise homecare services but give them a high level of autonomy to decide about their service based on their economic resources. • Homecare services are either provided by the municipalities themselves or contracted to private providers. The case manager at the local municipal authority approves type and extent of services based on individual assessments of needs. Services include both personal care, such as help with showering or help to dress, intake of medicines, help with eating and assistance with household tasks, for instance cleaning or grocery shopping. Assistance is provided around the clock. • More than half of Sweden’s municipalities have agreements with private homecare providers. Consequently, older individuals may choose between private, and municipality driven homecare providers. Both are regulated and followed up by the municipality [22]. If the potential client does not make an active choice, he or she will automatically be offered municipal homecare services. • In 2022 approximately 9% of Swedish citizens aged > 70 received homecare services authorized by municipalities [10, 23].	
• Many older individuals also receive home healthcare provided by the local primary care center. In Sweden healthcare is financed by taxes in 21 regions. Primary care is either provided by the Region or by private providers. Homecare and healthcare services are managed by different authorities with little or no collaboration [24]. • Since the 1990s there has been a significant change in the structure of the eldercare system in Sweden. Changes include shorter hospital stays, decrease in the numbers of beds in nursing homes [25], decrease of formal care and an increase of informal care such as support by family [26]. Furthermore, homecare workers report deteriorating working conditions with reduced time with the client, shortage of staff and rapid staff turnover [27].	

### Aim

The aim of this study was to understand if and how HCWs perceive they may contribute to the prevention of ED visits among home-dwelling older individuals. We did not present the participating HCWs with a predetermined definition of preventability. Instead, we listened to how they themselves perceived preventability.

## Method

### Design

A qualitative approach was used to explore HCWs views on how to prevent ED visits among home-dwelling older individuals. According to the aim of the study data were collected with semi-structured individual interviews [28] and were analysed using inductive thematic analysis [29]. We followed the consolidated criteria for reporting qualitative research (COREQ) [30] and supplementary file).

### Participants

Data for this study was collected as part of a larger interview study of HCWs´ experiences of homecare services [31]. Participants were invited to take part if they (1) had more than six months of working experience in homecare, (2) were interested in talking about their work in an one-hour interview.

We deemed a snowball sampling strategy as suitable as our prior experience showed that it is difficult to recruit HCWs for local development projects. However, the two first participants had no contact with HCWs outside their own workplace. To ensure geographical diversity and include urban as well as rural areas, we recruited participants through homecare managers. Accordingly, we contacted possibly eligible municipalities providing care themselves or through contracts to private providers. In total, we approached homecare providers in 8 different municipalities of which 6 (in 4 regions out of 21 in Sweden) agreed to participate. The responsible managers were informed about the study by telephone and email and asked interested HCWs to participate in the study. One week before the interview, HCWs who agreed to participate received information about the aim of the study. We informed them that they could contact the researcher (PBR) if they had any questions about the study and that they could decline further participation at any time during the interview. A total of 12 HCWs agreed to participate (Table 1).

**Table 1:** Characteristics of participants (*n* = 12)

Variables	n
Gender	
Male	1
Female	11
Age (years)	
Mean	44
Range	25–64
Born	
Sweden	9
Abroad	3
Nurse assistants^1^	6
Care assistant^2^	6
Work experience homecare services	
Mean	8 years
Range	1 year– 20 years
Care provider	
Municipality	5
Private company	7

^1^ Requires specialized education of 1–1/2 years at upper secondary school level

^2^ No formal education

### Data collection

Semi-structured interviews [28] were conducted between July and November 2022. The data collection started with two face-to-face interviews to pre-test the interview guide. The aim of the pre-test was to check the clarity of the questions. After this test, we broadened the questions to be more open-ended. All participants were interviewed over the telephone. One of the pre-test-interviews is included, whereas the other one was not audio-recorded and thus excluded. The managers offered the participating HCWs to conduct the interviews during working hours. All interviewees chose to answer the questions in their own homes due to the difficulty in finding a place at work to be undisturbed.

The interview guide with predefined topics was prepared to initiate and structure the interviews. To cover different aspects of perceived preventable ED visits the interviews started with broad open-ended questions, while the probing questions differ to give the participant the opportunity to elaborate and dwell on the aspects they chose as most relevant. Questions included were: What are trigger factors for ED visits in older home-dwelling individuals? Describe a case where a client had to visit the ED? Do you think something could have been done differently to prevent this client´s ED visit? Do you have any suggestions on how to prevent ED visits for older home-dwelling individuals in general? In Sweden, both publicly and privately ran homecare services commonly refer to older individuals receiving their assistance as clients, customers, or users, rather than patients. In this manuscript, we opt to use the term “clients” to characterize the older individuals receiving homecare services.

Data collection continued until information power was reached [32] i.e., when sufficient data had been collected to develop a robust and valid understanding of preventable ED visits. All interviews were audio-recorded and transcribed verbatim. The interviews ranged between 46 and 78 min with an average length of 54 min.

### Data analysis

Data were analysed using thematic analysis, a method for identifying, analyzing, and reporting patterns of meaning within data [29]. The analysis was completed manually. The following steps were taken in the analysis: (a) listening to the audio-recorded interviews and thoroughly reading the transcripts to develop familiarity and identifying patterns of meaning and potential interesting data; (b) the first author coded all transcripts, a process of dividing the text into meaningful units and giving each unit a name or a code. The coding process continued until all data was exhausted; (c) The first and the second author collated codes with similar content and grouped them into potential barriers and facilitators to prevent ED visits; (d) all potential barriers and facilitators were reviewed to find related patterns within and between them (Table 2). Barriers and facilitators considered to have a common origin were merged and organised into four categories: Accessibility to staff in PC; Information transfer between emergency departments and homecare services; Healthcare professionals´ attitudes towards competence and professionalism among homecare workers and Feelings of insecurity and anxiety among homecare clients. They were then merged under three headings: organisational, attitudes and client-related factors; d) all authors were involved in the process of refining, framing, and naming different categories of barriers and facilitators (Table 3); f) the report was produced, using quotes selected to illustrate each barrier and facilitator.

**Table 2:** Example of the process of data analysis

Extracts from interview (a)	Initial codes (b)	Collation of codes (c)	Category (d)	Aspect of category (d)
It had been almost three or four days without this person hadn´t eaten, and then my colleague decided that we should send her in by ambulance because this is no longer possible… It was more or less neglect, but nobody listened to us.	- We discover a lot with the older individuals - Listening to us	Facilitator to prevent ED visit	Healthcare professionals´ attitudes towards competence and professionalism among homecare workers	Perceived attitudes
Well, I won’t say unnecessary visit for the client, but sometimes I can think like this and it can depend a lot on us in the staff when clients perhaps in the summer are forced to seek emergency care because they are dehydrated. This is really something that we in the staff could have avoided.	- Generally, we need more knowledge	Barrier to promote ED visits		

### Rigour

Throughout the research process the authors maintained a reflective attitude and questioned any preconceptions. Our research group consists of individuals with different backgrounds (behavioural scientist/ nurse assistant, epidemiologist, general practitioner, clinical pharmacologist, and registered nurse) and perspectives. The authors´ experiences cover research and clinical work in eldercare as well as a broader health service perspective. To ensure credibility, the research team followed the guidelines of Braun and Clarke [29]. The initial analysis was performed by the first author and then discussed and validated by all authors. To increase dependability, all interviews were done by the same researcher (PBR) who encouraged every HCW to speak freely. To enhance confirmability, quotes are used to demonstrate the grounding of the findings in the data. All quotes were translated from Swedish to English. All authors iteratively reviewed the text before final approval. During the whole analytical process, several discussions with the five authors were conducted to enhance the trustworthiness of the findings.

## Results

The healthcare workers (HCWs) expressed diverse perspectives regarding their potential contributions to the prevention on ED visits among their clients. Participants acknowledged the challenges associated with preventing sudden deteriorations such as stroke, heart attack, and sepsis. Nonetheless, they cited instances where their knowledge of clients and nursing expertise could mitigate the need for ED visits. HCWs expressed a desire to be proactive in preventing ED visits but cited barriers stemming from both organisational factors and client-related issues, as well as perceived negative attitudes from primary care (PC) staff. Concurrently, they outlined potential solutions, referred to here as facilitators, to address these barriers (Table 3).

Furthermore, participants stated that gradual deteriorations in older individuals are commonplace. HCWs are often the first ones to observe such changes as they have regular, sometimes daily contacts with clients. Despite limited responsibility for medical care, HCWs feel compelled to respond to gradual deterioration that may cause increased discomfort or indicate that something more serious is about to happen. They expressed that early intervention is crucial to ensure that the client receives adequate help and support. For example, one HCW explained how small changes can have a big impact on the health of an older individual if they are not detected:

*“… what a wound looks like or if the patient is eating less, has lost weight. Such small things can be crucial information. I could go on and on, but this little thing can be quite significant. It could be due to a disease, maybe the person needs treatment”.* (Care Assistant, Interviewee 5)

**Table 3:** Barriers and facilitators for homecare workers´ ability to contribute to the prevention of ED visits among home-dwelling older individuals

Aspect	Category	Barriers	Facilitators
1. Organisational	Accessibility to staff in primary care	Absence of healthcare professional for information transfer or consultation	Collaboration and exchange of competence between homecare services and primary care - *Give nurse assistants in homecare more responsibility* - *In-house nurse* - *Medical advice during non-office hours* - *Increased preventive home healthcare*
	Information transfer between emergency departments and homecare services	Homecare workers do not receive discharge information from emergency departments	Discharge from hospitals in collaboration with homecare and with prompt follow up from primary care
2. Perceived attitudes	Healthcare professionals´ attitudes towards competence and professionalism among homecare workers	Primary care neither demands nor trusts the competence of homecare workers	Increased competence with training and support from primary care
3. Client-related	Feelings of insecurity and anxiety among homecare clients	Emergency department visits not always initiated for medical reasons	Flexible working conditions to enable homecare workers ensure a safe home situation

## Organisational

### Accessibility to staff in primary care

#### Barrier: absence of healthcare professional for information transfer or consultation

Participants explained that they act and try to inform the care manager or PC as soon as they notice a deterioration in the client’s health condition. However, they expressed concern that responses are often delayed, or that they may not receive any feedback at all if the information has reached the recipient. Meanwhile, there is a risk that the older person’s condition deteriorates further. They state that it is more difficult to get PCs to respond to subtle changes, such as loss of appetite, dehydration or increased lethargy, compared to a specific condition such as a suspected stroke. Participants expressed that gradual, stepwise deteriorations can escalate into emergencies if not addressed in time. HCWs described a high level of stress among PC professionals, which may explain not only delayed responses but also the reluctance of the PC professionals to evaluate a client. Instead, the advice is given to call an ambulance. Despite direct access to the PC practice via telephone, HCWs described unacceptable inertia in the healthcare system that prevents them from reaching out to staff with higher medical skills. For example, one HCW expressed how she feels they have to chase the staff from PC:.

*“You can never get hold of them (PC). I think there are three or four nurses. They never answer.… If you call the primary care practice at 11 a.m., they say - yes, leave your number, we’ll call you back at 5.30 p.m.… I have a telephone number to a doctor, but she usually doesn’t answer either.… Sometimes you have to try to catch a nurse who comes cycling down the street”.* (Care Assistant, Interviewee 1)

HCWs are often unsure whether the client needs immediate emergency admission. But since they cannot reach the PC for advice, they feel compelled to call an ambulance. Alternatively, they call the national healthcare information and advice service in Sweden which provides medical advice around the clock (1177^1^). One HCW described the urgent need of talking to qualified medical staff:

*“Since they couldn’t answer (PC), I just felt that I have to ask this now. I can’t walk away from him and leave it just like that, but I need to talk to someone so I know what to do. And then 1177 was the easiest, I thought. I have to solve it somehow”.* (Care Assistant, Interviewee 9)

All participants described shortcomings in communication and information transfer between homecare services and PC. HCWs are familiar with their clients and have a lot of information to share. They can therefore be an important channel to communicate the health status of their clients to the PC. However, they find that they rarely receive feedback on their reports, nor do they receive any information. Instead, HCWs get most of their information from the clients themselves. However, in the absence of medical information, they are not aware of the signs and symptoms they should be aware of. Due to poor information transfer between organisations and lack of communication channels, HCWs are unable to communicate changes in their clients´ well-being. One HCW stated:

*“If it was a family member who had contact with the primary care practice, we were not told anything unless it was something about medicine. But then it was usually the nurse who called and informed us, but we never found out anything from the primary care visits unless we had been there. So you didn’t really keep track of anyone in that way”.* (Care Assistant, Interviewee 2)

#### Facilitator: collaboration and exchange of competence between homecare services and primary care

*Assign nurse assistants in homecare more responsibility.* Participants emphasised their competence and skills, which could potentially reduce the burden on PC services. They suggested that colleagues trained nurse assistants should be given more responsibility for the management of uncomplicated medical conditions of a preventive nature. Examples included wound drainage, change of bandages, flushing or replacing urinary catheters, and fall accidents as these conditions, if not addressed, may cause an ED visit. Participants suggested that nurse assistants should perform triage to determine if an ambulance should be called or not. Furthermore, with access to basic medical equipment such as blood pressure monitors, pulse meters, and thermometers, nursing assistants would be able to perform basic medical assessments. In current homecare, medical equipment is not available unless the client holds it. Participants were convinced that the quality of homecare would increase significantly, and ED visits prevented if nursing assistants could perform initial assessments. A HCW stated that they have knowledge and should be given more responsibility and that they want to cooperate with PC services:

*“Home healthcare should listen to us a bit more, as we are knowledgeable. Sometimes you get a response that - well, that’s not your job.… we could have taken over a lot from home healthcare, such as taking blood samples, because we are nurse assistants, we can do that, but also to make the job easier for them. So more cooperation and that they are happy to put more responsibility on us nurse assistant so we can work with the medical part as well. We’re good at dressing wounds, but sometimes we’re told - but you shouldn’t do that because you can’t do it, you’re not allowed to do it but we have to do it-, even though we can”.* (Nurse Assistant, Interviewee 4)

*In-house nurse.* One participant raised a good example of the implementation of an in-house nurse (employed in the homecare organisation) which has proven beneficial as it allows for quick and easy access to healthcare. The close collaboration between the nurse and HCWs promotes a continuous dialog, facilitates the exchange of observations and information, and provides timely advice when needed. The participant noted that this approach not only improves the quality of care but also reduces unnecessary ED visits. A HCW gave an example on how they cooperate with the nurses:

*“I always go to the nurses. Is there something that has happened, he is not the same? And then we can go home to the patient together and make an assessment.…. for example if someone has fallen and has an open wound and you don’t need to go to the emergency room for that wound. We can take care of the wound. Or you take a picture and send it to the nurse, and then we discuss it.”* (Care Assistant, Interviewee 5).

*Medical advice during non-office hours.* During evenings and weekends, participants reported feeling particularly vulnerable because the PC is closed. In one municipality HCWs can call 1177 and be connected to a medical team. This on-call team makes home visits for urgent medical needs. Participants considered this approach is valuable because they receive medical advice on time and during non-office hours. A HCW stated:

*“We need to have a dialogue with someone because we can’t take on and assess something that we don’t have the authority to do. Just like in the hospital, there is some kind of back-up for us in the homecare service.”* (Nurse Assistant, Interviewee 7).

*Increased preventive home healthcare.* Participants felt that PC does not provide enough preventive care for older individuals living at home, and that preventive PC is essential to minimize the number of ED visits. Moreover, that hospitalisations could be avoided by home-based care for specific health issues, such as oxygen therapy for breathing difficulties. Proactive treatment and management of such conditions might improve the individual´s overall well-being, reduce anxiety, promote a sense of safety at home, and reduce the likelihood of seeking ED care in non-urgent situations. One HCW described that older individuals may consult the ED because of lack of equipment:

*“When it comes to individuals with multiple diseases, one disease can trigger another. COPD, for example, is such a thing, that you feel that you can’t breathe and you have nothing to treat it with. Oxygen is needed but there is no one who can come and give oxygen, so you have to go on an hour and a half trip to the emergency room”.* (Care Assistant, Interviewee 6)

### Information transfer between emergency departments and homecare services

#### Barrier: Homecare workers do not receive discharge information from emergency departments

HCWs receive information through a computerized system if and for how long a client is hospitalized, and the expected time for discharge. However, if a client is discharged on the same day, which is a common occurrence according to HCWs, there is no information available from the computerized system. Consequently, HCWs will remain uninformed about whether the client has returned home or not. Therefore, they want to receive information from the ED even when the client is sent home on the same day. Immediate homecare assistance may be required as the client may be tired and confused after an ED visit. A HCW described the consequences of being unaware of discharge from ED as follows:

*“There are no available beds and they (ED) can say they are done with the treatment, but do you understand? Send home an old person at 2–3 in the night. Home to loneliness, no one there. I think it’s terrible. It’s feels as if it costs too much money to have them there”.* (Care Assistant, Interviewee 10)

Discharge from ED without any information or explanation from ED to homecare may cause anxiety and even result in readmission to ED. One HCW stated:

*“That older individuals are sent home too quickly (from the ED). Then they are back again just one or two days later…. And then they are sent home, sometimes on the same day without having done anything. Then you haven’t succeeded, I think”.* (Care Assistant, Interviewee 6)

#### Facilitator: Discharge from hospital in collaboration with homecare and by prompt follow up from primary care

The participants expressed that the concept of safe discharge^2^ to home rarely works in practice for clients who are discharged on the same day. They state that PC should immediately follow up older individuals who are discharged. Otherwise, early readmission is likely. Safe home discharge relies on close cooperation between homecare services and PC. However, homecare must be informed about the clients’ progress within the care trajectory. One HCW perceived that older individuals are often discharged prematurely for questionable reasons:

*“ You don’t want to have patients in hospital for too long, because that costs a lot of money, and there is an agreement between the municipality and the region, that they should be send home as soon as medically possible. What is medically possible? It is not always easy to investigate”.* (Care Assistant, Interviewee 6)

## Perceived attitudes

### Healthcare professionals´ attitudes towards competence and professionalism among homecare workers

#### Barrier: primary care neither demands nor trusts the competence of homecare workers

HCWs do not feel listened to when they report changes in an older person’s health to PC. This lack of attention negatively affects the clients´ health and increases their anxiety. Sometimes HCWs have no choice but to refer the client to the ED because their observations and concerns have been ignored despite an untenable situation at home. One HCW described that she had to call an ambulance because the PC did not listen to their observations:

*“It had been almost three or four days without this person hadn´t eaten, and then my colleague decided that we should send her in by ambulance because this is no longer possible… It was more or less neglect, but nobody listened to us”.* (Care Assistant, Interviewee 2)

Participants expressed that, in case of deteriorated health, they often have to advocate on behalf of their clients to ensure immediate care or appointments for PC assessments.

HCWs experienced situations where ED visits could have been prevented if their observations had been taken seriously. Despite raising their concerns, participants experienced that nurses and physicians in PC do not act timely. One HCW described how they have to be assertive in their dealings with the PC:

*“I go to the client every day, I see that something is not right, then they (PC) can say, “We’ll wait for the rest of the week, keep an eye out”. In some cases you have to give in, but if you see that there is something special that is very untypical, then I say no, we will not wait. And then they come. So you have to be able to stand your ground in this job. Sometimes they (PC) say “No, but it’s probably not that bad”. But it is…. I wish they would listen from the beginning and acknowledge that you meet the clients every day and you can see if there is anything strange”.* (Nurse Assistant, Interviewee 8)

#### Facilitator: increased competence with training and support from primary care

Participants proposed a collaborative work approach between HCWs and PC staff where they receive guidance or internal training from PC nurses on how to manage challenging situations. They believed that this approach not only can prevent ED visits but also create a holistic view of older individuals’ situation in home and facilitate teamwork between the different care organisations. One HCW described it like this:

*“I think it’s the nurses (in PC) who should provide training. It is quite important to know what to do in different situations. How do you proceed if someone suddenly lies on the floor lifeless? I think many individuals can get very scared and panic…”* (Nurse Assistant, Interviewee 10).

Although all participants had experience working with older people, they stressed the urgent need for improved medical knowledge among the entire HCW organisation and its staff. Some participants stated that this is particularly important as medical needs among older home-dwelling individuals have increased, and as it is difficult to separate an individual’s medical needs from regular social care. HCWs work with many inexperienced colleagues who lack basic medical knowledge. Consequently, these colleagues have difficulty preventing ED visits, initiating necessary visits and distinguishing between the two. Participants recall several incidents where clients should have been admitted to the ED but were denied by colleagues lacking competence. Conversely, there were cases where an ED visit could easily have been avoided if appropriate measures had been taken, such as in case of dehydration or hypoglycemia. One example from a HCW reads as follows:

*“Well, I won’t say unnecessary visit for the client, but sometimes I can think like this and it can depend a lot on us in the staff when clients perhaps in the summer are forced to seek emergency care because they are dehydrated. This is really something that we in the staff could have avoided”.* (Nurse Assistant, Interviewee 10)

## Client-related

### Feelings of insecurity and anxiety among older home-dwelling individuals

#### Barrier: emergency department visits not always initiated for medical reasons

Compared to one decade ago, participants experienced that frailty among older homecare clients has increased, and that clients suffer from a higher number of multiple long-term conditions including mental illness. Participants stated that anxiety is a major reason why their clients visit the ED. In these cases, the clients often call for an ambulance themselves. HCWs reflected that clients who frequently attend the ED due to anxiety rarely receive any help and are often sent home the same day, perpetuating a vicious cycle where anxiety often increases and causes further ED visits. Participants believed that there are insufficient resources to address older individual’s anxiety. A HCW described this vicious cycle as follows:

*“The client herself has called an ambulance, even though she might not really have needed it; she might just have needed someone to come and talk to her, someone who knows her problems. But instead she has gone to the emergency room and they send her home immediately. She continues as usual and a few days go by and then she calls again and goes to the emergency room”.* (Nurse Assistant, Interviewee 9)

HCWs reported that they were unaware of services that may assist clients in these situations, aside from calling the clinic for psychiatric emergency care. However, clients rarely reach out to them directly, perhaps because they are unaware of their own anxiety. Instead, anxious clients may suspect that their chest pain or heart palpitations have somatic reasons. HCWs observed that PC mainly offers drug treatment against anxiety but lacks resources to provide other help such as counselling support. Consequently, participants´ attempts to arrange psychological help within PC are often fruitless. An example of this is described by an HCW:

*“I go to a client who feels very anxious and feels that there are small animals in the bed or snakes and such…. I have contacted home healthcare and I have documented. I tried to calm this client… No, they (PC) can’t help that person because it’s not medical.”* (Nurse Assistant, Interviewee 4).

#### Facilitator: flexible working conditions to enable homecare workers ensure a safe home situation

HCWs believed that increased social interaction could diminish anxiety and prevent many ED visits and wanted more time to engage in these activities. They were confident in their ability to reduce older individuals’ anxiety as they have good client knowledge and thus could provide the support clients need to feel secure. They observed that many older individuals are lonely and suffer from their loneliness. One HCW described the importance of social interaction for older home-dwelling individuals:

*“I think it’s the social interaction that needs to be increased. Because sometimes that is what is needed to avoid a doctor’s visit. It can be small or simple things that are needed to avoid more care… It’s probably one of the most important things, that you are listened to. Social interaction is incredibly important. I would say that we are good at managing anxiety…”* (Care Assistant, Interviewee 6).

## Discussion

This study aimed to increase the understanding of whether and how HCWs perceive that they could contribute to the prevention of ED visits among older home-dwelling individuals. The results show that HCWs reflect on how they can play a crucial role in preventing ED visits among the individuals they care for. Thanks to their familiarity and regular contact with homecare recipients, HCWs considered that they could observe early deterioration in older individual’s health, which often occurs gradually over time. Small deteriorations are potentially treatable in PC through early intervention [33]. Still, HCWs experienced that many barriers hinder them from transferring their knowledge and actively contributing to the prevention of ED visits among older individuals. Barriers but also suggestions for improvement were organized into three categories: organisational factors such as availability of PC staff; communication and information transfer between organisations as well as attitudes towards HCWs as co-workers; and client-related factors.

Direct communication, collaboration, and coordination of care between HCWs and PC are crucial to provide high-quality care for older home-dwelling individuals [9, 19, 34]. Lack in communication and information transfer between care providers is a major risk to the safety of older people [19, 35]. Nevertheless, we find that PC often are inaccessible to HCWs and there are no effective communication channels. HCWs have difficulties sharing their knowledge about clients but also receiving information on individuals’ medical needs. This is in line with an earlier study stating that HCWs rarely received feedback from healthcare or information about a client’s health condition [19]. Consequently, HCWs are not able to keep track of a client’s medical history or treatment plans which makes it difficult to communicate urgent concerns to PC. On the other hand, PC lacks important patient information limiting the ability to act on medical issues before they become urgent [19]. Participants describe that they can offer valuable insights into an older person’s daily routines and living environment, which may enhance PC´s understanding of the person’s overall well-being. In turn, PC staff may provide medical expertise, guidance on treatment plans, and specialized interventions to address complex health conditions. Both Scandinavian and international literature describes that communication and information transfer between HCWs and PC are indispensable to ensure care coordination, increase awareness of changes in the client´s condition and thus avoid ED visits [9, 19, 35, 36].

Compared to a decade ago, older individuals in many countries who receive care at home seem to be more vulnerable and have a higher prevalence of long-term conditions [1–4, 37]. The high need of medically qualified HCWs has not been met and staff experience that they often are faced with decisions and tasks that exceed their resources and competence. HCWs are expected to handle practical situations that traditionally lie beyond the scope of homecare [37]. The distinction between homecare and home healthcare has become increasingly blurred. It may be challenging to differentiate between tasks associated with general well-being and activities in daily living (homecare) and those related to medical treatment (healthcare), as they often are interconnected and intertwined. For instance, assisting an older person with support stockings is a homecare task involved in the clients´ daily living and dressing. However, HCWs attentiveness to signs of increased swelling or pain in the client’s legs can be crucial for ongoing healthcare and overall well-being. The participating HCWs desire geriatric education from PC nurses which may strengthen collaboration and empower HCWs.

In a complex healthcare system, care should be planned in close collaboration between professionals from different organisations and in partnership with older individuals and relatives [38]. However, professionals rarely succeed in providing such coordinated care [39]. One example experienced from HCWs in this study refers to the discharge from EDs which may occur without adequate information. Contrary to PC, HCWs lack access to the medical record and are not able to track a client´s progress within the care trajectory. As a result, HCWs have to rely on oral discharge information from clients or their relatives. Participants observed that numerous older individuals are not admitted to a hospital ward but rather sent home immediately after the ED visit which hampers “safe discharge to home” (see footnote 2 in result). In case of discharge from ED prompt visits from HCWs and medical follow-up by PC may be required. The successful discharge of older individuals from ED to home is a process that relies not only on HCWs´ commitment and skills but collaboration between HCWs and PC staff [36]. Possible solutions may be telehealth and secure messaging platforms which may enhance communication and facilitate real-time information exchange [40]. However, to ensure the usability of such technical solutions HCWs must be involved in their development. Guidance on how to address ethical challenges about what information about a client HCWs and PCs are permitted to see and share, one suggestion is to approach clearer guidance from the healthcare regions and the municipalities in which information sharing is allowed and even encouraged [19].

Loneliness is common among older individuals and may cause illness and disease [41]. According to participants, older individuals may interpret loneliness and consecutive anxiety as somatic symptoms that often triggers ED visits. While PC often suggests treating anxiety with drugs, participants believe that supportive measures such as meaningful conversations with clients are more important to meet clients´ needs and prevent anxiety-driven ED visits. HCWs themselves are used to engage in meaningful conversations with clients, as they have regular interactions and a deep understanding of the person’s situation [42, 43]. Participants considered themselves pivotal in identifying and mitigating mental health conditions in older individuals. Nonetheless, they face significant time constraints and lack flexibility in their daily schedule, which hampers their ability to fully address these concerns [44].

The participants´ experiences are in line with earlier research reporting a high prevalence of mental illness among older individuals [41–43, 45]. In Sweden, every third woman and every fifth man over the age of 77 report problems with anxiety [45]. Moreover, depressive disorders are frequent in older individuals and may be considered as a public health problem. Still, mental illness is seldom acknowledged and treated; according to the Public Health Authority in Sweden knowledge about occurrence, symptoms and treatment is limited both among older individuals and their relatives but also among important actors in healthcare [45]. Consequently, only few older individuals receive adequate and timely help. Besides PC, HCWs should be recognized as trustful collaborators who are important for preventive work and early detection of symptoms of mental illness in older individuals [43].

HCWs suggest several measures to alleviate anxiety and thereby prevent potential ED visits. First, HCWs have a trustful relationship with their clients, enabling comfortable discussions about fears and concerns [43, 46]. Second, older individuals may feel anxious due to uncertainty or lack of understanding about their health condition or care process. HCWs can play a crucial role in alleviating clear and accurate information about their treatment plans, and any upcoming procedures [43]. This may empower the client, give a feeling of control, and reduce their anxiety. Third, HCWs may ensure that daily activities such as meals and medication follow a consistent schedule. Older individuals may benefit from such structured routines which increase their feeling of security. Finally, HCWs can help older individuals maintain social connections with friends, family, and community groups by arranging visits or connecting them with support networks. These social interactions contribute to a sense of belonging and reduce feelings of isolation and anxiety [41, 43].

For HCWs to be able to contribute to the prevention of ED visits, the study results suggest that PC centers gain from the expertise of HCWs by having registered nurses at PCs provide comprehensive clinical education and integrate them as associated to their teams. The quality of care for frail older individuals at home could be significantly improved if HCWs were offered medical training, were integrated into a collaborative team alongside nurses and doctors at PC and were supported by registered nurses. This, in turn, may change any negative attitudes of the PC towards the competences of the HCW. Furthermore, the healthcare regions and the municipalities should address issues regarding what information HCWs and PC staff are permitted to access and share about clients. There should be clear guidelines on information sharing, with encouragement for appropriate sharing practices.

### Strengths and limitations

The qualitative approach we used is valuable for providing rich descriptions of complex phenomena such as the prevention of ED visits. Telephone interviews are frequently overlooked in qualitative research and are often portrayed as a less appealing option compared to face-to-face interviews. Research on the method has found that there is no evidence for the critics of loss of contextual and nonverbal information, probing, and the interpretation of responses [47]. Instead, telephone interviews might offer respondents a relaxed environment conducive to disclosing sensitive information, and there is no clear evidence indicating lower data quality [47]. In this study, we felt that the HCWs could talk openly and freely about their work and the generated data gave a rich and nuanced picture of HCWs´ views. However, this study reflects views of skilled and experienced HCW with in mean 8 years of working experience (Table 1) which may not be representative with regards to the high staff turnover in homecare services [27]. Other views may be held by temporary staff with less education and experience which is common in many homecare organisations in Sweden, this may consequently restrict the transferability of the findings. Another potential limitation was the sampling method with involvement of managers in recruiting HCWs. In this case, there is a power differential between managers and HCW with the risk that HCW felt forced to participate and/or be afraid to talk about negative experiences. Moreover, the managers may have selected HCWs they knew would be more positive towards their work. Consequently, there is a possibility we did not manage to capture the full range of HCWs negative experiences.

The research team´s different experiences and competencies from the medical and sociological fields have contributed constructively to reflexivity and the interpretation of data. The trustworthiness of the findings is strengthened by the use of multiple researchers engaged in each of the analytical phases.

## Conclusion

HCWs perceived that they have an important role in the management of health problems of older individuals living at home but feel they represent an untapped resource in preventing ED visits. HCWs reported that they are well placed to identify early signs of health and social problems in individuals because they are in continuous contact with clients. They believe that their knowledge is useful in preventing minor health problems from turning into ED visits. HCWs have detailed knowledge about their clients and therefore have important information to share with PC staff. However, information sharing requires access to PC staff, regular communication, and supportive trustful collaborations. HCWs believed that robust coordination and communication between PC, ED, and homecare organisations as well as proactive care can increase the overall well-being of older people living at home and prevent ED visits.

### Electronic supplementary material

Below is the link to the electronic supplementary material.

Supplementary Material 1

## Data Availability

The data that support the findings of this study are available but restrictions apply to the availability of these data, which were used under license for the current study, and so are not publicly available. Data are however available from the authors upon reasonable request and with permission of corresponding author.

## Footnotes

1 1177 is a national healthcare information and advice service in Sweden that provides medical advice 24/7, guidance, and information to the general public. The service is available to anyone living in Sweden and is operated by the Swedish County Councils. 1177 is staffed by registered nurses who have experience in PC and can provide advice on a wide range of health issues. The nurses may also refer individuals to other healthcare services, such as emergency departments or primary healthcare clinics, as needed.

2 Safe discharge from hospital for older individuals aims ensuring that the person can return to their home environment safely after being hospitalized. This means that staff from the municipality and from the local primary healthcare center in collaboration prepare a discharge plan matching the needs of older individuals of extra support after leaving the hospital.

## Abbreviations

ED: Emergency department
HCW: Homecare workers
PC: Primary care
