# Supplementary material for: Homecare workers - an untapped resource in preventing emergency department visits among older individuals? A qualitative interview study from Sweden
Source: BMC Geriatr. 2024 Apr 18;24:350. doi: 10.1186/s12877-024-04906-5 (PMC11027288; doi:10.1186/s12877-024-04906-5)
Supplement: Supplementary file 1 — Supplementary Material 1 [file 12877_2024_4906_MOESM1_ESM.docx]

**Manuscript:** **Home care workers - an untapped resource in preventing emergency department visits of older people? A qualitative interview study from Sweden**

**Consolidated criteria for reporting qualitative studies (COREQ): 32-item checklist**

Developed from:

Tong A, Sainsbury P, Craig J. Consolidated criteria for reporting qualitative research (COREQ): a 32-item checklist for interviews and focus groups. *International Journal for Quality in Health Care*. 2007. Volume 19, Number 6: pp. 349 – 357

| **No. Item** | **Guide questions/description** | **Reported** |
| --- | --- | --- |
| **Domain 1: Research team and reﬂexivity** | | |
| *Personal Characteristics* | | |
| 1. Inter viewer/facilitator | Which author/s conducted the interview or focus group? | Page 11, heading Riogour and page 32, heading Authors´ contributions. |
| 2. Credentials | What were the researcher’s credentials? E.g. PhD, MD | Page 32, heading Authors´ contributions. |
| 3. Occupation | What was their occupation at the time of the study? | Page 10, heading Rigour and page 32, heading Authors´ contributions. |
| 4. Gender | Was the researcher male or female? | Page 32, heading Authors´ contributions. |
| 5. Experience and training | What experience or training did the researcher have? | Page 30, heading Strenghts and limitations, and page and 32, heading Authors´ contributions. |
| *Relationship with participants* | | |
| 6. Relationship established | Was a relationship established prior to study commencement? | No, page 6, heading: Paticipants.  . |
| 7. Participant knowledge of the interviewer | What did the participants know about the researcher? e.g. personal goals, reasons for doing the research | Page 31, heading: Ethics approval and consent to participate. |
| 8. Interviewer characteristics | What characteristics were reported about the interviewer/facilitator? e.g. Bias, assumptions, reasons and interests in the research topic | Page 30, heading Strenghts and limitations. |
| **Domain 2: study design** | | |
| *Theoretical framework* | | |
| 9. Methodological orientation and Theory | What methodological orientation was stated to underpin the study? e.g. grounded theory, discourse analysis, ethnography, phenomenology, content analysis | Page 6, heading Design and page 9-10 heading Data analysis. |
| *Participant selection* | | |
| 10. Sampling | How were participants selected? e.g. purposive, convenience, consecutive, snowball | Page 6, heading Participants. |
| 11. Method of approach | How were participants approached? e.g. face-to-face, telephone, mail, email | Page 8, heading Data collection. |
| 12. Sample size | How many participants were in the study? | Page 6, heading Participants, and Table 1. |
| 13. Non-participation | How many people refused to participate or dropped out? Reasons? | Page 6, heading Participants. |
| *Setting* | | |
| 14. Setting of data collection | Where was the data collected? e.g. home, clinic, workplace | Page 8, heading Data collection.  . |
| 15. Presence of non-participants | Was anyone else present besides the participants and researchers? | No, page 8, heading Data collection. |
| 16. Description of sample | What are the important characteristics of the sample? e.g. demographic data, date | Page 7, Table 1 characteristics of participants. |
| *Data collection* | | |
| 17. Interview guide | Were questions, prompts, guides provided by the authors? Was it pilot tested? | Page 8, heading Data collection. |
| 18. Repeat interviews | Were repeat interviews carried out? If yes, how many? | No |
| 19. Audio/visual recording | Did the research use audio or visual recording to collect the data? | Page 8, heading Data collection. |
| 20. Field notes | Were ﬁeld notes made during and/or after the interview or focus group? | No |
| 21. Duration | What was the duration of the interviews or focus group? | Page 8, heading Data collection. |
| 22. Data saturation | Was data saturation discussed? | Page 8, heading Data collection  (information power). |
| 23. Transcripts returned | Were transcripts returned to participants for comment and/or correction? | No |
| **Domain 3: analysis and ﬁndings** | | |
| *Data analysis* | | |
| 24. Number of data coders | How many data coders coded the data? | 2, page 9, heading Data analysis. |
| 25. Description of the coding tree | Did authors provide a description of the coding tree? | Table 2, page 9-10. |
| 26. Derivation of themes | Were themes identiﬁed in advance or derived from the data? | Derived from data, page 9, heading Data analysis. |
| 27. Software | What software, if applicable, was used to manage the data? | No, data were analysed manually, page 9, heading Data analysis. |
| 28. Participant checking | Did participants provide feedback on the ﬁndings? | No. |
| *Reporting* | | |
| 29. Quotations presented | Were participant quotations presented to illustrate the themes/ﬁndings? Was each quotation identiﬁed? e.g. participant number | Yes, page 11-24, heading Results. |
| 30. Data and ﬁndings consistent | Was there consistency between the data presented and the ﬁndings? | Yes, page 11-24, heading Results. |
| 31. Clarity of major themes | Were major themes clearly presented in the ﬁndings? | Yes, page 11-24, heading Results and Tabel 3, page 13. |
| 32. Clarity of minor themes | Is there a description of diverse cases or discussion of minor themes? | Yes, in the results and the discussion. |
